# In vitro screening of a FDA approved chemical library reveals potential inhibitors of SARS-CoV-2 replication

**DOI:** 10.1038/s41598-020-70143-6

**Published:** 2020-08-04

**Authors:** Franck Touret, Magali Gilles, Karine Barral, Antoine Nougairède, Jacques van Helden, Etienne Decroly, Xavier de Lamballerie, Bruno Coutard

**Affiliations:** 1 Unité Des Virus Emergents (UVE: Aix Marseille Univ, IRD 190, INSERM 1207, IHU Méditerranée Infection), 13005 Marseille, France; 2 Aix-Marseille Univ, INSERM U1068, CNRS UMR7258, Institut Paoli-Calmettes, CRCM, Marseille, France; 3 Institut Français de Bioinformatique (IFB), UMS 3601-CNRS, Université Paris-Saclay, Orsay, France; 4 Aix-Marseille Univ, INSERM, Lab. Theory and Approaches of Genome Complexity (TAGC), Marseille, France; 5 AFMB UMR 7257, Aix-Marseille Université, CNRS, Marseille, France

**Keywords:** Drug discovery, SARS-CoV-2

## Abstract

A novel coronavirus, named SARS-CoV-2, emerged in 2019 in China and rapidly spread worldwide. As no approved therapeutics exists to treat COVID-19, the disease associated to SARS-Cov-2, there is an urgent need to propose molecules that could quickly enter into clinics. Repurposing of approved drugs is a strategy that can bypass the time-consuming stages of drug development. In this study, we screened the PRESTWICK CHEMICAL LIBRARY composed of 1,520 approved drugs in an infected cell-based assay. The robustness of the screen was assessed by the identification of drugs that already demonstrated in vitro antiviral effect against SARS-CoV-2. Thereby, 90 compounds were identified as positive hits from the screen and were grouped according to their chemical composition and their known therapeutic effect. Then EC50 and CC50 were determined for a subset of 15 compounds from a panel of 23 selected drugs covering the different groups. Eleven compounds such as macrolides antibiotics, proton pump inhibitors, antiarrhythmic agents or CNS drugs emerged showing antiviral potency with 2 < EC50 ≤ 20 µM. By providing new information on molecules inhibiting SARS-CoV-2 replication in vitro, this study provides information for the selection of drugs to be further validated in vivo. Disclaimer: This study corresponds to the early stages of antiviral development and the results do not support by themselves the use of the selected drugs to treat SARS-CoV-2 infection.

## Introduction

Human coronaviruses (HCoVs) are enveloped positive-stranded RNA viruses belonging to the *Nidovirales* order that are mostly involved in gastrointestinal and respiratory tract infections. Among them, severe acute respiratory syndrome (SARS) and Middle East respiratory syndrome (MERS) CoVs that emerged in 2002 and 2013 respectively, have been associated with severe human illnesses, such as severe acute respiratory distress syndromes^1^. In December 2019, a new coronavirus (SARS-CoV-2) has emerged in the city of Wuhan and quickly spread around the world. SARS-CoV-2 causes in human a viral infection, named COVID-19, which is associated in some patients with severe respiratory diseases and significant mortality rates, in particular in elderly populations^2^. While an unknown fraction (most probably a majority) of infected people remain pauci- or asymptomatic, some require hospitalization, sometimes in intensive care units, which has jeopardised health systems during peak pandemic periods. In such a context, vaccines would represent great tools to prevent or limit virus spread. However, vaccine development is a long and uncertain process and COVID-19 vaccines will most probably not be concretely available for mass usage, at least during the first wave of the disease. Accordingly, the availability of efficient antiviral drugs would be of utmost interest for the treatment of infected patients and possibly for preventive use. Regrettably, the current and unprecedented outbreak of SARS-CoV-2 occurs in an unprepared world, with no firmly established identification of active molecules against beta-CoVs^3^. There is thus an urgent necessity to provide here and now therapeutic solutions to limit viral infection. As the timeframe for a conventional drug development is unrelated to the immediate needs, repurposing of drugs originally developed for other viral infections or therapeutic uses is likely the fastest way to enter clinics. This fast track drug development and validation lead to the initiation of numerous clinical trials for the treatment of COVID-19^4^ but there is still a need to expand the number of possible drug candidates to treat COVID-19 and/or evaluate possible drug combinations to potentiate the antiviral effects^5^. Whereas the number of clinical trials cannot be extensively multiplied, libraries of “old” drugs can be screened in vitro in medium- to high-throughput assays to significantly reduce the number of candidate molecules. In addition, screening of approved drugs can also pave the way for medicinal chemistry programs^6^. Screenings of drugs to be repositioned against SARS-CoV, MERS-CoV or other viruses already showed their relevance for the selection of antivirals active at least in vitro^7–9^. In this study, we screened the 1,520 approved and off-patent drugs of the PRESTWICK CHEMICAL LIBRARY in a SARS-CoV-2 infection cell-based assay. From this in vitro screening, ninety drugs were identified as inhibitors of the viral cytopathic effect at 10 μM. Fifteen hits, selected from different therapeutic classes, were then confirmed by EC50 and CC50 determination.

## Material and methods

### Chemical library

The PRESTWICK CHEMICAL LIBRARY (hereafter named PCL) is a library of 1,520 off-patent small molecules, mostly approved drugs (FDA, EMA and other agencies). The compounds are provided at a concentration of 10 mM in 100% DMSO.

### Cell lines

VeroE6 (ATCC CRL-1586) and Caco-2 (ATCC HTB-37) cells were grown in minimal essential medium (THERMOFISHER SCIENTIFIC, Waltham, USA) with 7 0.5% heat-inactivated fetal calf serum (FCS), at 37 °C with 5% CO_2_ with 1% penicillin/streptomycin (PS, 5000 U mL^−1^ and 5000 µg mL^−1^ respectively; THERMOFISHER SCIENTIFIC, Waltham, USA) and supplemented with 1% non-essential amino acids (THERMOFISHER SCIENTIFIC, Waltham, USA).

### Virus strain

SARS-CoV-2 strain BavPat1 was obtained from Pr Drosten through EVA GLOBAL (https://www.european-virus-archive.com/). To prepare the virus working stock, a 25 cm^2^ culture flask of confluent VeroE6 cells growing with MEM medium with 2.5% FBS (THERMOFISHER SCIENTIFIC, Waltham, USA) was inoculated at MOI 0.001. Cell supernatant medium was harvested at the peak of infection and supplemented with 25 mM HEPES (SIGMA, St Louis, USA) before being stored frozen in small aliquots at − 80 °C. All experiments were conducted in BSL3 laboratory.

### Antiviral screen

One day prior to infection for the antiviral screening 5 × 10^4^ VeroE6 cells were seeded in 100 µL assay medium (containing 2.5% FCS) in 96 well plates. The next day, a single dilution of each compound of the PCL at 10 µM final concentration was added to the cells (25 µL/well, in 2.5% FCS-containing medium). Six virus control wells were supplemented with 25 µL medium (positive controls hereafter named vc) and eight cell control wells were supplemented with 50 µL of medium (negative controls, hereafter named nc). Two internal well controls of viral inhibition were added, corresponding to the addition of 10 µM arbidol (SIGMA, St Louis, USA) in the infected cell culture (arbidol controls, hereafter named arb). After 15 min, 25 µL of a virus mix diluted in 2.5% FCS-containing medium was added to the wells at MOI 0.002.

Three days after infection, cell supernatant media were discarded and CellTiter-Blue reagent (PROMEGA, Fitchburg, USA) was added following the manufacturer’s instructions. Plates were incubated for 2 h prior recording fluorescence (560/590 nm) with a Tecan Infinite 200Pro machine (TECAN, Zurich, Switzerland). From the measured OD_590nm_, the Inhibition Index was calculated as follows: first, cell viability for compounds, vc and arb were calculated: (OD_590nm_ value/mean OD_590nm_ of nc) × 100. For vc and arb, mean cell viability were calculated. Then all cell viabilities were normalized by subtracting mean vc. cell viability of the 96 well plates. Finally, Inhibition index was calculated as follows: Inh. Index = normalized cell viability of the compound/normalized cell viability of arb in the same 96 well plate.

### Data analysis of the screening and selection of the hits

The data analysis was performed following two strategies. First, each drug with Inhibition index value above 1 was considered as a hit. A statistical analysis was also applied to the screening in order to assess the statistical significance of the viability. The raw OD values were normalized by computing the log2-ratio between treated cells and the median of the nc of the same plate.

$$ L_{m,i} = log2\left( {\frac{{OD_{m,i} }}{{OD_{nc,i} }}} \right) $$

where $OD_{m,i}$ is the optical density of cells treated with molecule *m* on plate i, $OD_{nc,i}$ is the median OD of the 8 negative control wells on the same plate, and $L_{m,i}$ is the log-ratio of treatment *m* on plate i. A relative viability $V_{m,i}$ is defined on a scale where 0 and 100 respectively correspond to the median values of the vc and nc of the same plate.

$$ V_{m,i} = 100 \cdot \frac{{L_{m,i} - L_{vc,i} }}{{L_{nc,i} - L_{vc,i} }} $$

This viability is standardized by computing a z-score based on robust estimators (median for central tendency and inter-quartile range for the dispersion.

$$ z_{m,i} = \left( {V_{m,i} - \widetilde{{V_{i} }}} \right)\frac{{Q3_{N} - Q1_{N} }}{{Q3_{i} - Q3_{i} }} = \left( {V_{m,i} - \widetilde{{V_{i} }}} \right)\frac{1.349}{{Q3_{i} - Q3_{i} }} $$

where $\widetilde{{V_{i} }}$ is the median relative viability of plate i, whereas Q1 and Q3 denote the first and third quartiles of the measures on the plate (*i* subscript) and in the standard normal distribution (*N* subscript). We compute a normal P value, which is adjusted by deriving a false discovery rate (FDR), which indicates the expected proportion of false discoveries among the molecules declared positive [].

### EC50 and CC50 determination

One day prior to infection, 5 × 104 VeroE6 cells were seeded in 100µL assay medium (containing 2.5% FCS) in 96 well plates. The next day, seven twofold serial dilutions of compounds (0.6–40 µM, in triplicate) were added to the cells (25 µL/well, in assay medium). Four virus control wells were supplemented with 25 µL of assay medium. After 15 min, 25 µL of a virus mix diluted in medium was added to the wells. The amount of virus working stock used was calibrated prior to the assay, based on a replication kinetics, so that the replication growth is still in the exponential growth phase for the readout as previously described^10,11^. Four cell control wells (i.e. with no virus) were supplemented with 50 µL of assay medium. On each plate a control compound (Remdesivir, BLDPHARM, Shanghai, China) was added in duplicate with seven twofold serial dilutions (0.16–20 µM, in duplicate). Plates were incubated for 2 days at 37 °C prior to quantification of the viral genome by real-time RT-PCR. To do so, 100 µL of viral supernatant was collected in S-Block (QIAGEN, Hilden, Germany) previously loaded with VXL lysis buffer containing proteinase K and RNA carrier. RNA extraction was performed using the Qiacube HT automat and the Cador Pathogen 96 HT kit following manufacturer instruction. Viral RNA was quantified by real-time RT-qPCR (EXPRESS One-Step Superscript qRT-PCR Kit, universal Invitrogen using 3.5 µL of RNA and 6.5 µL of RT qPCR mix and standard fast cycling parameters, i.e*.*, 10 min at 50 °C, 2 min at 95 °C, and 40 amplification cycles (95 °C for 3 s followed by 30 s at 60 °C). Quantification was provided by four 2 log serial dilutions of an appropriate T7-generated synthetic RNA standard of known quantities (10^2^ to 10^8^ copies). RT-qPCR reactions were performed on QuantStudio 12K Flex Real-Time PCR System (APPLIED BIOSYSTEMS, Waltham, USA) and analyzed using QuantStudio 12 K Flex Applied Biosystems software v1.2.3. Primers and probe sequences, which target SARS-CoV-2N gene, were: Fw: GGCCGCAAATTGCACAAT; Rev: CCAATGCGCGACATTCC; Probe: FAM-CCCCCAGCGCTTCAGCGTTCT-BHQ1. The 50% and 90% effective concentrations (EC50, EC90; compound concentration required to inhibit viral RNA replication by 50% and 90%) were determined using logarithmic interpolation as previously described^11^. For the evaluation of the CC50 (the concentration that reduces the total cell number by 50%), the same culture conditions were set as for the determination of the EC50, without addition of the virus, and cell viability was measured using CellTiter Blue (PROMEGA, Fitchburg, USA) as previously described for the screening. CC50 was determined using logarithmic interpolation. All data obtained were analyzed using GraphPad Prism software version 7.0 (GRAPH PAD software Inc, California, USA). All graphical representations were also performed on GraphPad Prism software version 7.0 (https://graphpad-prism.software.informer.com/7.0/).

### Drugs validation in Caco-2 cells

Antiviral activity was evaluated as described above for EC50 and CC50 determination in Caco-2 cells (ATCC HTB-37), except that compounds were evaluated in duplicate at two finale concentrations 5 and 10 µM. All data obtained were analyzed using GraphPad Prism software version 7.0 (GRAPH PAD software). All graphical representations were also performed on GraphPad Prism software version 7.0(GRAPH PAD software Inc, California, USA, https://graphpad-prism.software.informer.com/7.0/).

## Results and discussion

We developed an HTS SARS-CoV-2 replication inhibition assay based on the measurement of the cell viability 3 days after cell infection with a MOI of 0.002. Prior to the screening, we evaluated the antiviral effect of arbidol, a broad-spectrum antiviral compound that blocks the viral entry of many enveloped viruses^12^. In our experimental conditions, we demonstrated that 10 µM arbidol limits the SARS-CoV-2 infection leading to 70–90% cell viability, with EC50 of 10.7 µM. This compound was next used as plate-specific reference in order to calculate the inhibition index (Inh. Index).

**Figure 1:**
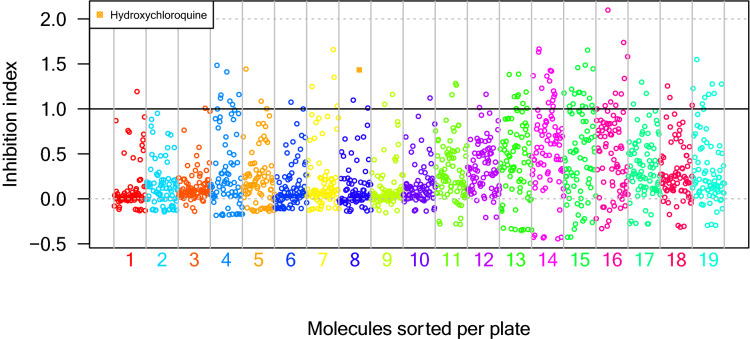
Screening of 1,520 clinically approved compounds from PRESTWICK CHEMICAL LIBRARY and hits selection**.** The black dot line represents the threshold for positive hit compounds. Numbers associated with colors represent the plate numbers where the compounds are located.

We next screened the PRESTWICK CHEMICAL LIBRARY (PCL) composed of 1,520 approved drugs at a final concentration of 10 µM. The cell viability was determined and we calculated the relative value of inhibition potency compared to arbidol. Among the 1,520 compounds of the PCL, 90 hit molecules showed equal or higher inhibition than arbidol with an Inh. Index ≥ 1 (5.85% positive hits) (Fig. 1; Supp Table 1). In addition, a statistics-based filter was applied in order to limit effect of inter-plaques screen variation and select compounds with a FDR > 0.05. This criterion allowed the selection of 56 hits (Supp Table 2), 14 among them overlap with the list of hits having an Inh. Index ≥ 1 and are depicted in Table 1 as the highest confident hits. Nevertheless, as the thresholds for the selection remain arbitrary, the raw data for each compound of the PCL are presented in the supplemental data, allowing the scientific community to further analyse the results and possibly rescue molecules of interest.

**Table 1:** Inhibition index and detailed description of the 14 highest confident hit compounds.

ID	Chemical name	Reported therapeutic effect	Inhibition index
01F08	Benoxinate hydrochloride	Local anesthetic	1.19
03G08	Clemizole hydrochloride	Antihistaminic, antibiotic, antineoplastic	1.01
05A10	Tacrine hydrochloride	CNS stimulant	1.44
06D11	Epiandrosterone	Anabolic	1.07
07B04	Omeprazole	Antiulcer	1.25
07G07	Pregnenolone	Anabolic, anti-inflammatory	1.66
07G09	Chloroquine diphosphate	Anti-inflammatory, antimalarial, antiprotozoal	1.35
07H07	Mirtazapine	Antidepressant	1.03
08D06	Exemestane	Antineoplastic	1.10
08E11	Hydroxychloroquine sulfate	Antimalarial, anti-inflammatory, antirheumatic	1.43
08H03	Dipivefrin hydrochloride	Antiglaucoma	1.01
09D04	Opipramol dihydrochloride	Antidepressant	1.05
09F04	Promazine hydrochloride	Antipsychotic	1.16
10G08	Merbromin disodium salt	Antiseptic, antibacterial	1.12

**Table 2:** Antiviral activity of selected hit compounds and control compounds.

µM	EC50	EC90	CC50	SI
Azithromycine	2.12	8.65	> 40	> 19
Spiramycin	7.95	10.45	> 40	> 5
Omeprazole	17.06	38.01	> 40	> 2.35
Oxprenolol hydrochloride	20.22	> 40	> 40	> 2
Hydroxy-chloroquine	4.17	25.49	> 40	> 10
Clemizole hydrochloride	23.94	38.23	> 40	> 1.7
Alprostadil	5.39	62.40	> 40	> 7.4
Dolutegravir	22.04	42.81	> 40	> 1.8
Sulfadoxine	35.37	45.11	> 40	> 1.13
Opipramol dihydrochloride	5.05	5.97	> 40	> 7.9
Quinidine hydrochloride	5.11	> 40	> 40	> 7.8
Vonoprazan	38.58	41.01	> 40	> 1
Exemestane	7.51	9.86	> 40	5.3
Dyclonine hydrochloride	10.00	> 40	> 40	> 4
Spiperone	2.49	13.10	> 40	> 16
Arbidol	10.7	15.2	> 40^a^	> 3.7
Remdesivir 7 exp	1.67 ± 0.59	2.53 ± 0.67	nd	nd

All value are in µM.

*EC50* 50% inhibition, *EC90* 90% inhibition, *CC50* 50% cytotoxicity, *SI* selectivity index, *nd* not determined.

^a^From Ref.^33^.

For a better readability, the ninety hits displaying an Inh. Index ≥ 1 were then organised in 12 groups by structural similarity and/or therapeutic class (depicted in Supp. Table 1) including steroids, prostaglandins, proton pump inhibitors, antiretrovirals, antibacterial drugs, cardiovascular drugs, opioids plus non-opioid CNS drugs, neuromuscular-blocking drugs, respiratory system drugs, allergy medications, antiparasitic drugs and few unrelated drugs.

Among these 90 hits, we identified drugs that previously demonstrated to inhibit in vitro the SARS-CoV2 replication. Accordingly, Chloroquine and Hydroxychloroquine^13–16^ were shown to limit in vitro SARS-CoV-2 replication with an Inh. Index of 1.35 and 1.43 respectively. In addition to chloroquine derivatives, two other hits are also currently evaluated in different clinical trials, namely Darunavir^17^ and Azythromycine^18^.

**Figure 2:**
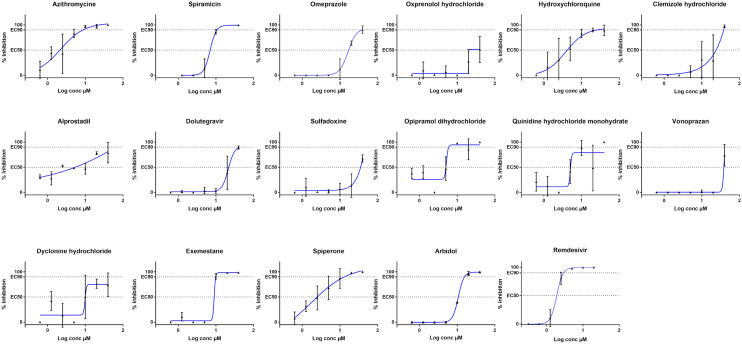
Dose response curves of selected hit compounds. EC50: 50% inhibition, EC90: 90% inhibition. Compounds concentrations are presented in log scale for logarithmic interpolation. Dose response curves were generated using GraphPad Prism software version 7.0 (https://graphpad-prism.software.informer.com/7.0/).

In a second stage, we consolidated the provided screening results by performing EC50 values determination on a set of selected compounds. Whereas the screening relied on the quantification of cytopathic effect (CPE) using CellTiter Blue providing qualitative information on the viral infection, EC50 determination were based on the quantification of the viral genome by Real-Time RT-PCR^11^. For this assay, a broad-spectrum antiviral, namely Remdesivir, was used as a validation control since it displays an EC50 of 1.6 µM (Table 2, Fig. 2), a value in agreement with previously published data^14^.

Then, we aimed at determining the 50% inhibition dose (EC50), 50% cytotoxicity dose (CC50) and selectivity index (SI: CC50/EC50) for a panel of 23 drugs selected to cover the 12 different groups previously described. Regarding the drugs selection, the most efficient and confident hits were prioritized. Some hits were arbitrary removed after inspection of their initial therapeutic use or strong side effects (i.e. ophthalmic treatment, topical administration, teratogenic effect …). EC50 values could be determined for 15 drugs out of 23 (Table 2, Fig. 2). Indeed, a poor antiviral effect (EC50 ≥ 40 µM) was observed for Darunavir as recently described^19^, Levalbuterol, Olmesartan, Ambrisentan, Cefamandole, Formoterole and Ranolazine in this orthogonal assay. As these compounds were selected from an initial screening assay based on SARS-CoV-2-mediated CPE inhibition, we cannot exclude that these compounds have no antiviral effect but rather protect the cells from CPE. In addition, Artenimol was rejected as it showed toxicity at 20 µM concentration, in the same range of the antiviral effect. Interestingly and despite a lack of activity at 40 µM, Candesartan, Olmesartan and Ambriesartan are interesting hits because they interfere with angiotensin pathways. These pathways play a key role in virus entry and the SARS-CoV2 Spike protein is known to bind to the cellular Angiotensin Converting Enzyme 2 receptor (ACE2)^20,21^. Moreover a clinical trial will be conducted with Telmisartan^22^, another Angiotensin II Receptor Blocker (ARB) derivative. Moreover recent studies have shown that smokers have lower risk of contracting COVID-19^23–25^, and one explanation could be the interference between the nicotine which downregulates the ACE2 receptor^26^.

Among the 15 hits for which EC50 values were determined (Table 2, Fig. 2), two of the highest antiviral activity were obtained for Azithromycine (EC50 = 2.12 µM) and Hydroxychloroquine (EC50 = 4.17 µM) that were selected for clinical trials^18^. For these two compounds, the dose response curves have at least two values above EC90, which demonstrates a clear in vitro activity as already described for Hydroxychloroquine^13–15,27^. EC90 of Azithromycine is 8.65 µM and the one of Hydroxychloroquine is 25.49 µM, these values reflect that the concentration of Hydroxychloroquine has to be significantly higher than Azithromycin to totally block the viral replication. Vonoprazan and Sulfadoxine show a moderate activity with EC50 above 30 µM. As titration started at 40 µM in our assays, one cannot conclude at this stage if this EC50 reflects a cytotoxic effect of the drug or a true antiviral effect. Spiperone, a dopaminergic D2 antagonist, already identified as an antiviral molecule against the human pathogenic Polyomaviruses^28^ presents the second highest SI with a value of 16. The next three most efficient drugs are Opipramol dihydrochloride (SI ˃ 7.9), a tricyclic antidepressant (TCA) also described to block the entry of Ebola virus and Marburg virus in HTS assays (Supp. Table 1 and bioassay results from PubChem); Quinidine hydrochloride (SI ˃ 7.8), an antiarrhythmic drug that also displaying antimalarial effect and described to inhibit HCV and HCMV replication in HTS assays (Supp. Table 1 and bioassay results from PubChem); and Alprostadil (SI ˃ 7.4), a prostaglandin known as cardiovascular drug which displays a variety of pharmacologic actions and has been in clinical trials for respiratory distress syndrome^29^ (Supp. Table 1). The remaining 9 drugs out of 15 are described in Table 2 and show 1.0 < SI < 5.3. Two of them, Omeprazole (SI ˃ 2.3) and Vonoprazan (SI ˃ 1.0), are proton pump inhibitors (PPIs) used as antiulcer agents. Omeprazole is specifically of interest because it is massively used and well tolerated. It has been demonstrated to increase the pH of endosomial/golgian pathway either by inhibiting ATPase proton pomp, or by buffering the pH. We can thus expect that such endosomial pH modification would limit the processing of the Spike protein by endosomal proteases and, in turn, bloke the virus entry mediated by membrane fusion process.

**Figure 3:**
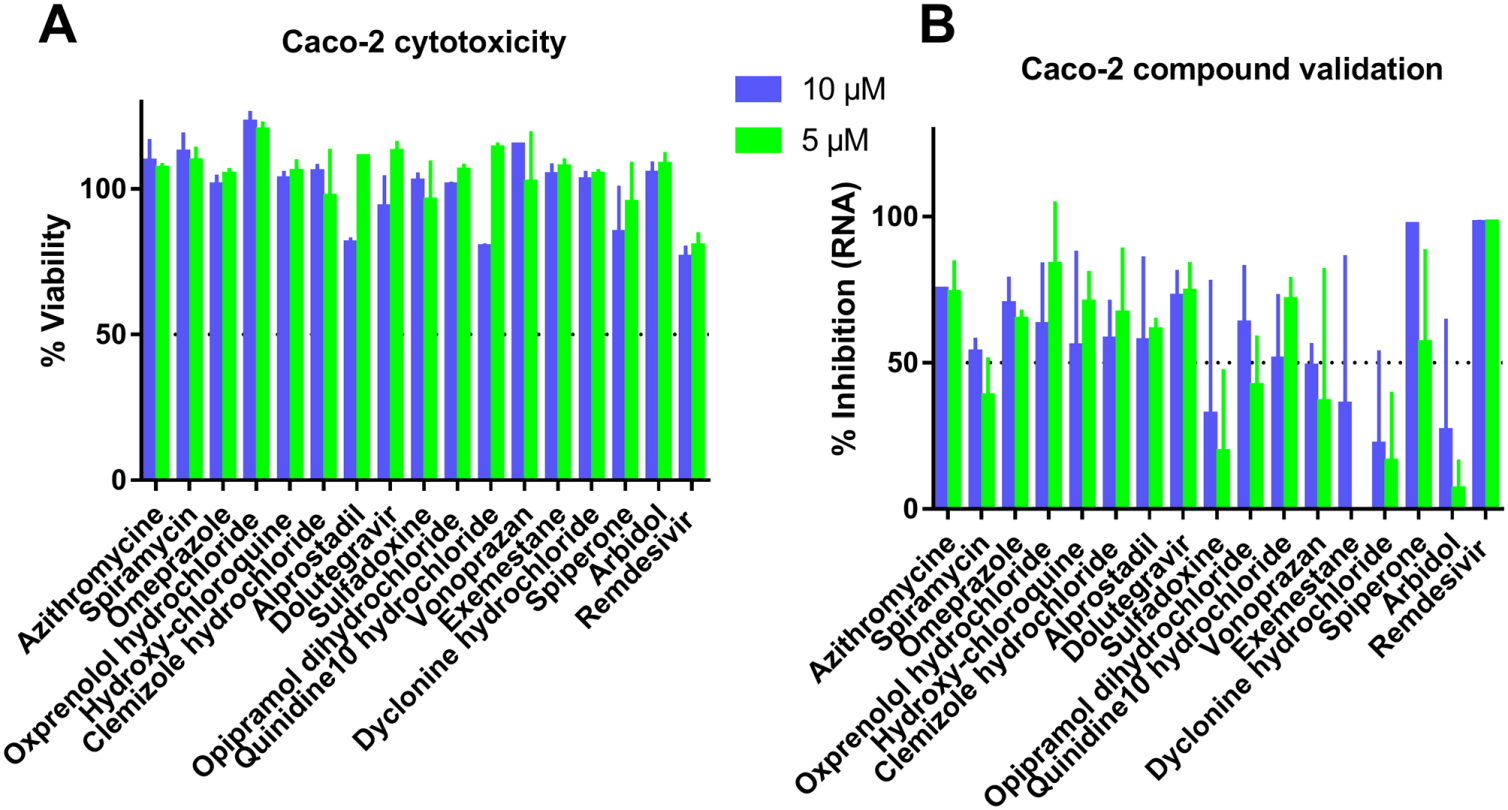
Antiviral activity validation of the 17 compounds presented in Table 2 in Caco-2 cells at 5 and 10 µM. (**A**) Cytotoxicity of compounds in Caco-2 evaluated by the measure of cells viability. (**B**) Antiviral activity determined by the viral replication inhibition measurement in Caco-2. Graphical representations were generated using GraphPad Prism software version 7.0 (https://graphpad-prism.software.informer.com/7.0/).

To assess the reliability of the antiviral effect observed for t the 17 drugs whose EC50 values were determined (Table 2 and Fig. 2), we validated their antiviral effect in Caco-2 cells at two concentrations, 5 and 10 µM (Fig. 3). Whereas Remdesivir shows 100% replication inhibition at 5 and 10 µM, the effect of Arbidol is weaker than the one observed in VeroE6. For most of the selected drugs, inhibition can be observed at both concentrations, except for Sulfadoxine, Exemestane and Dyclonine hydrochloride that show an Arbidol-like result. This double evaluation in two different cell lines remains of interest since compounds such as Sofosbuvir have been reported to display cell type-dependent efficacy^30^.

The main advantage of drug repurposing is to by-pass pre-clinical tests and save time in an epidemic context. As these drugs are not originally developed and optimised against infectious agents, it is not surprising that the most efficient drugs display EC50 above 2 µM against SARS-CoV-2 replication and are thus less potent than known antivirals such as Remdesivir. However, most of these compounds being already approved as drugs, they demonstrate a low cytotoxicity effect, and even with high EC50, their Selectivity Index remains of interest. At this stage, it is essential to lead a high number of drug candidates as far as possible into the drug development process. Indeed, even efficient in vitro candidates, such as Hydroxychloroquine or Azythromycin, showing EC50 values lower than the concentration observed in lung tissues, can demonstrate no antiviral effect in a macaque model^31^.

In conclusion, we developed an infected cell-based assay to select antivirals against SARS-CoV-2. The screening of the approved drugs allowed identifying a set of 15 molecules showing inhibition on SARS-CoV-2 in vitro replication. Interestingly, the antiviral activity of some compounds identified in our studies has been recently confirmed in other cell based screening on SARS-CoV-2^32^. Some of these experimentally selected candidates, with EC50 at the 2–20 µM range may provide information to guide the choice for downstream experiments and validation in small animal models, initiate medicinal chemistry projects to find more potent derivatives, or evaluate in vitro the combination of drugs to potentiate effects of drugs with originally moderate benefit.

## Supplementary information

Supplementary information
