# Supplementary material for: In vitro screening of a FDA approved chemical library reveals potential inhibitors of SARS-CoV-2 replication
Source: Sci Rep. 2020 Aug 4;10:13093. doi: 10.1038/s41598-020-70143-6 (PMC7403393; doi:10.1038/s41598-020-70143-6)
Supplement: Supplementary file 1 — Supplementary information [file 41598_2020_70143_MOESM1_ESM.pdf]

In vitro screening of a FDA approved chemical library reveals potential inhibitors of SARS-CoV-2 replication

Franck Touret[1*], Magali Gilles[1], Karine Barral[2], Antoine Nougairède[1], Jacques van Helden[3,4]
Etienne Decroly[5], Xavier de Lamballerie[1], and Bruno Coutard[1*]

Supplementary Information

Supplementary Table 1

The table cannot be implemented in the final PDF and is thus available here :

https://amubox.univ-amu.fr/s/pMbp2ibiQTGKfHi

Supplementary Table2

The table cannot be implemented in the final PDF and is thus available here :

https://amubox.univ-amu.fr/s/P5CqTw3soJW4PpE

Raw Data

The table cannot be implemented in the final PDF and is thus available here :

https://amubox.univ-amu.fr/s/LFNP8PLZtRLk9Cm
